# Spermidine delays aging in humans

**DOI:** 10.18632/aging.101517

**Published:** 2018-08-06

**Authors:** Frank Madeo, Didac Carmona-Gutierrez, Oliver Kepp, Guido Kroemer

**Affiliations:** 1 Institute of Molecular Biosciences, NAWI Graz, University of Graz, Graz, Austria; 2 BioTechMed Graz, Graz, Austria; 3 Metabolomics and Cell Biology Platforms, Gustave Roussy Cancer Campus; Villejuif, France; ^4^Equipe 11 Labellisée Ligue Nationale contre le Cancer, Centre de Recherche des Cordeliers, Paris, France; ^5^INSERM, U1138, Paris, France; 6 Université Paris Descartes/Paris V, Paris, France; 7 Université Pierre et Marie Curie/Paris VI, Paris, France; 8 Pôle de Biologie, Hôpital Européen Georges Pompidou, AP-HP; Paris, France; 9 Department of Women's and Children's Health, Karolinska University Hospital, Stockholm, Sweden

**Keywords:** autophagy, health span extension, cancer

**Summary:** External supply of the natural polyamine spermidine can extend life span in model organisms including yeast, nematodes, flies and mice. Recent epidemiological evidence suggests that increased uptake of spermidine with food also reduces overall, cardiovascular and cancer-related mortality in humans. Here, we discuss the possible mechanisms of this intriguing spermidine effect.

Polyamines including spermidine play an essential role in intermediate metabolism. Since they are synthesized by higher eukaryotic cells, they are not vitamins. However, the levels of polyamines are profoundly influenced by their external supply, either by oral ingestion with different food items or by the intestinal microbiota that can synthesize polyamines as well [1].

Our groups have shown over the past decade that supplementing spermidine by adding it to culture media (as we did for the yeast *Saccharomyces cerevisiae*, the nematode *Caenorhabditis elegans* and the fruit fly *Drosophila melanogaster*) or to the drinking water (as we did for the rodent *Mus musculus*) is sufficient to extend longevity and to improve health span at multiple levels [2,3]. Thus, in mice, the supplementation was able to suppress the age-related decline in cardiovascular function (as measured at 24 months of age) and increased overall longevity by approximately 10% [3]. Of note nutritional uptake of spermidine and spermine but not putrescence could be linked to improved cardiovascular health and autopsies performed at death did not reveal any significant effect of spermidine on the incidence of cancer, suggesting that the reduction of cardiovascular morbidity was not compensated by an increase in malignancies [3]. Rather, in mice, spermidine postpones the manifestation cancer upon oncogenic stimuli [4,5].

Moreover, fragmentary evidence suggests that spermidine can also delay neurodegeneration, both in non-mammalian model organisms [6] and in mouse models [7,8].

The molecular and cellular mechanisms through which spermidine delays age-related disease and death have been elucidated to some extent. Indeed, spermidine can act as an inhibitor of the acetyl transferase activity of E1A-associated protein p300 (where E1A = adenovirus early region 1A), best known as EP300 [9]. EP300 act as an endogenous inhibitor of autophagy by acetylating lysine residues within multiple proteins that are involved in autophagy-regulatory or autophagy-executing circuitries [1,10]. As a result, the inhibition of EP300 by spermidine (which competes with the acetyl group donor acetyl coenzyme A) stimulates autophagy [9] Autophagy is required for the anti-aging effect of spermidine as indicated by the fact that genetic inhibition of autophagy (by knockout or knockdown of essential autophagy-relevant genes) abolishes the longevity-extending effects of spermidine on yeast, worms and flies [11]. Moreover, in mice, deletion/depletion of essential autophagy genes in myocardial or cancer cells reduces the beneficial effects of spermidine on cardiovascular disease and cancer, respectively [3,4]. Autophagy is a major mechanism of cellular adaptation to stress, as well as the most important pathway for the turnover of cytoplasmic structures including whole organelles, thus facilitating the rejuvenation of important portions of the cell. For this reason, autophagy has a vast anti-aging potential to the point that most if not all behavioural, nutritional, pharmacological or genetic manipulations that extend longevity require autophagy to be efficient [12–14].

Until now the literature on the longevity-enhancing effects of spermidine has been limited to model organisms. Now, two prospective population-based studies (summarized in the same paper) report for the first time that nutritional spermidine uptake is also linked to reduced overall, cardiovascular and cancer-related mortality in humans [15]. Both studies were based on the use of food questionnaires that allowed to calculate for each individual the nutritional uptake of polyamines including spermidine. Importantly, high spermidine uptake constituted an independent favourable prognostic parameter for reduced mortality, meaning that this variable predicted a reduced incidence of death even after correction for possible confounding factors such as age, body mass index, consumption of alcohol or aspirin, diabetes, metabolic syndrome, physical activity, sex, socioeconomic status and even dietary quality, supporting the idea that spermidine might indeed be causally involved in a reduction of overall morbidity and mortality [15].

In addition to the aforementioned epidemiological results, there are further, though admittedly indirect arguments in favour of a health-improving role for spermidine in human health. Thus, spermidine has been classified as a “caloric restriction mimetic” that has broad health-promoting effects due to its capacity to induce similar biochemical changes as does caloric restriction [16]. Second, the proximal pharmacological target of spermidine is the same as that of salicylic acid, the active metabolite or aspirin (both inhibit EP300 by competing for the binding of acetyl coenzyme 1) [17], knowing that aspirin is probable the one single drug that has the broadest positive impact on human mortality from cardiovascular and malignant disease [18].

**Figure 1:**
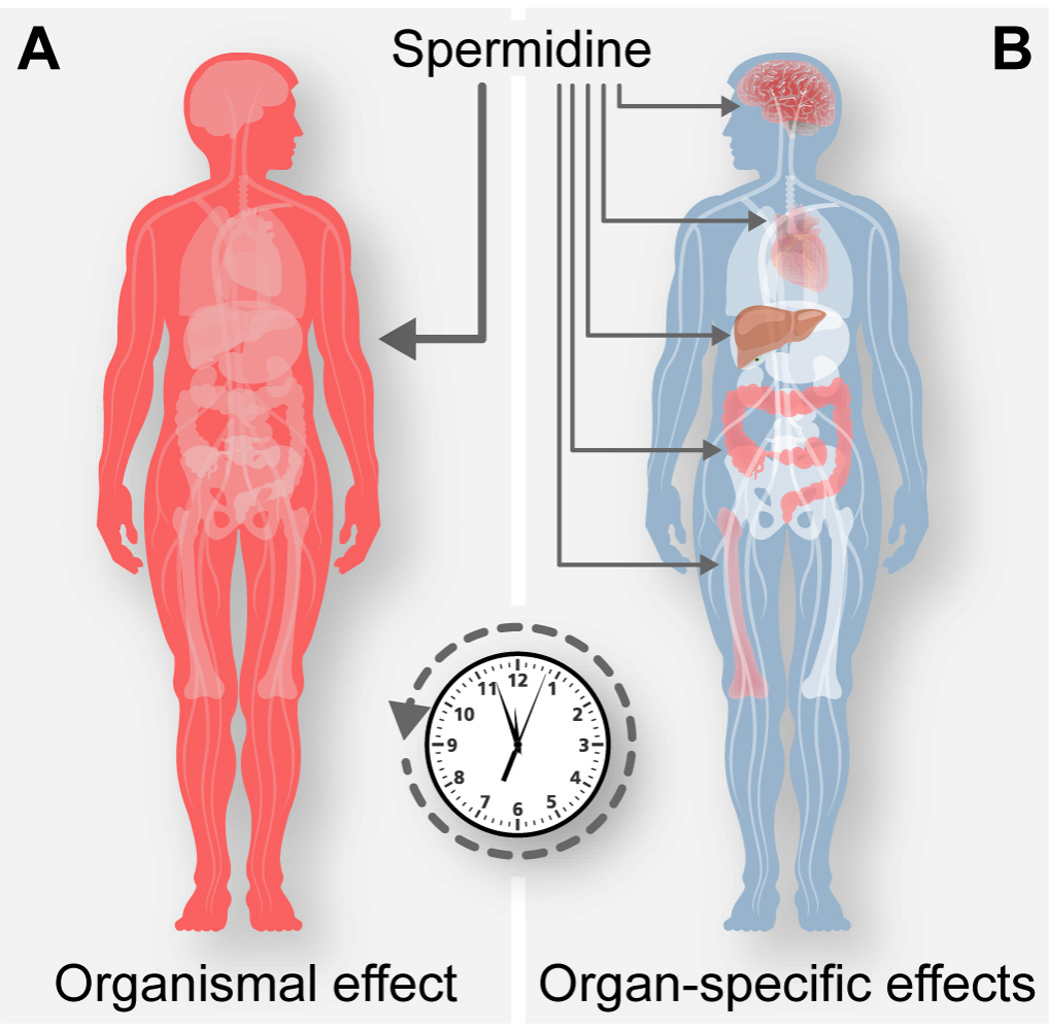
**Possible mechanisms of spermidine-mediated rejuvenation.** Spermidine may counteract the general clock of aging, by a global effect on cellular fitness (**A**), or may exert specific effects on multiple organ systems engaged in for example cardiovascular function, anticancer immune surveillance or neurodegeneration and thereby reducing the incidence of the major age-related diseases (**B**).

The fine mechanism through which spermidine (and aspirin) have such a broad effect on human health have not yet been fully elucidated. Based on current knowledge, these agents may slow down the general clock of the aging process, for instance by a global effect on cellular fitness, thereby mediating a pleiotropic effect on all aging-related diseases. The health-improving effects of aspirin have been initially attributed to its capacity to inhibit thrombocyte aggregation (via inhibition of cyclooxygenase) and hence to act as an anti-coagulant. Since spermidine has not been reported to have similar anti-coagulant activity, we prefer the hypothesis that aspirin may mediate its broad pro-health effects via the inhibition of EP300. As an alternative, yet non-exclusive mechanism, the natural EP300 inhibitor spermidine and its pharmacological equivalent aspirin may both act on different yet distinct cell types including stem cell compartments and differentiated cells engaged in cardiovascular function (cardiac muscle cells, endothelial cells, pericytes, small vessel myocytes…), anticancer immune surveillance (cancer and immune cells) or neurodegeneration (neuronal and glial cells) to reduce the incidence of the major age-related diseases (Figure 1). Future research must elucidate the molecular pathways on which spermidine acts to identify actionable targets that may be used for the treatment and prevention of age-related diseases.
